# Pressure-Induced Superconductivity of the Quasi-One-Dimensional Organic Conductor (TMTTF)_2_TaF_6_

**DOI:** 10.3390/ma15134638

**Published:** 2022-07-01

**Authors:** Miho Itoi, Toshikazu Nakamura, Yoshiya Uwatoko

**Affiliations:** 1 Physics Section, Division of Natural Sciences, Nihon University School of Medicine, Itabashi 173-8610, Japan; 2 Institute for Molecular Science, Okazaki 444-8585, Japan; t-nk@ims.ac.jp; 3 Institute for Solid State Physics, The University of Tokyo, Kashiwa 277-8581, Japan; uwatoko@issp.u-tokyo.ac.jp

**Keywords:** one-dimensional organic conductor, superconductivity, high-pressure measurement

## Abstract

We investigated the superconductivity of (TMTTF)_2_TaF_6_ (TMTTF: tetramethyl-tetrathiafulvalene) by conducting resistivity measurements under high pressure up to 8 GPa. A cubic anvil cell (CAC) pressure generator, which can produce hydrostatic high-pressure, was used for this study. Since the generalized temperature-pressure (*T*-*P*) diagram of (TMT*C*F)_2_*X* (*C* = Se, S, *X*: monovalent anion) based on (TMTTF)_2_PF_6_ (*T*_CO_ = 70 K and spin-Peierls: SP, *T*_SP_ = 15 K) was proposed by Jérome, exploring superconductivity states using high-pressure measurement beyond 4 GPa has been required to confirm the universality of the electron-correlation variation under pressure in (TMTTF)_2_*X* (TMTTF)_2_TaF_6_, which has the largest octahedral-symmetry counter anion TaF_6_ in the (TMTTF)_2_*X* series, possesses the highest charge-ordering (CO) transition temperature (*T*_CO_ = 175 K) in (TMTTF)_2_*X* and demonstrates an anti-ferromagnetic transition (*T*_AF_ = 9 K) at ambient pressure. A superconducting state in (TMTTF)_2_TaF_6_ emerged after a metal-insulator transition was suppressed with increasing external pressure. We discovered a superconducting state in 5 ≤ *P* ≤ 6 GPa from *T*_c_ = 2.1 K to 2.8 K, whose pressure range is one-third narrower than that of *X* = SbF_6_ (5.4 ≤ *P* ≤ 9 GPa). In addition, when the pressures with maximum SC temperatures are compared between the PF_6_ and the TaF_6_ salts, we found that (TMTTF)_2_TaF_6_ has a 0.75 GPa on the negative pressure side in the *T*-*P* phase diagram of (TMTTF)_2_PF_6_.

## 1. Introduction

Since the discovery of the first organic superconductivity in (TMTSF)_2_PF_6_ (tetramethyl-tetraselenafulvalene-hexafluorophosphate), quasi-one-dimensional (Q1D) organic conductors (TMT*C*F)_2_*X* (*C* = Se and S, *X* = monovalent anion) have been extensively investigated because electronic-correlations related to spin, charge, and dimensionality generate various types of ground states [1,2,3,4,5]. In the (TMT*C*F)_2_*X* crystal*,* face-to-face TMT*C*F molecules align perpendicularly along the *a*-axis. The hybridization of p-electrons on Se or S atoms in the TMT*C*F molecules leads to strong one-dimensional conductivity, and the 1D-TMT*C*F chain is well-separated by the monovalent anion *X* layer. The (TMT*C*F)_2_*X* system has a 3/4 filling band structure [6]. Their flexible-molecular packing consequently produces multihued ground states, which change from AFM (anti-ferromagnetism) I-SP (spin-Peierls), AFM II (commensurate SDW), and incommensurate SDW (spin density wave) to an SC (superconductivity) phase at low temperatures, by controlling the superposed electron density in both inter-and intra-chains using chemical and the applied pressures [1]. For more than three decades, the generalized phase diagram has been extended by efforts in synthesizing TMTTF salts with *centrosymmetric* (*cs*) (*X* = Br, I, PF_6_, AsF_6_, SbF_6_, NbF_6,_ and TaF_6_) and *non-centrosymmetric* (*ncs*) anions (*X* = BF_4_, ClO_4_ and ReO_4_). Simultaneously, many scientists have attempted to clarify the ground state changes of (TMTTF)_2_*X* by conducting pressure measurements, and the validity of the temperature-pressure (*T***-***P*) diagram corresponding between the chemical pressure and the external pressure has been confirmed. In particular, the high-pressure investigation exceeding 4 GPa has revealed the existence of the superconductivity phase in the (TMTTF)_2_*X* series [2,3,7,8,9,10].

The emergence of the superconducting phase in the (TMTTF)_2_*X* series is understood as a crossing over anti-ferromagnetic fluctuation. (TMTTF)_2_Br is the first superconductor in TMTTF series (*T*_C_ = 0.8 K@ 2.6 GPa) [11], whose ground state shifts from AFM II (*T*_AF_ = 15 K, C-SDW: commensurate) to SDW (I-SDW: incommensurate) by external pressure [12,13,14]. The SDW transition temperature decreases due to the imperfect nesting of Fermi surfaces by applying external pressure. The SDW phases observed in (TMTSF)_2_*M*F_6_ (*M* = P, As, Sb) were explained by the electron correlation and two-dimensionality with mean-field theory [15]. As it stands that the SC phase in (TMT*C*F)_2_*X* always neighbors the SDW, there are many reports about the exotic superconductivity properties of (TMT*C*F)_2_*X*, for instance, the anisotropic SC revealed by high magnetic field measurements [16,17] and by muon spin rotation [18].

**Figure 1:**
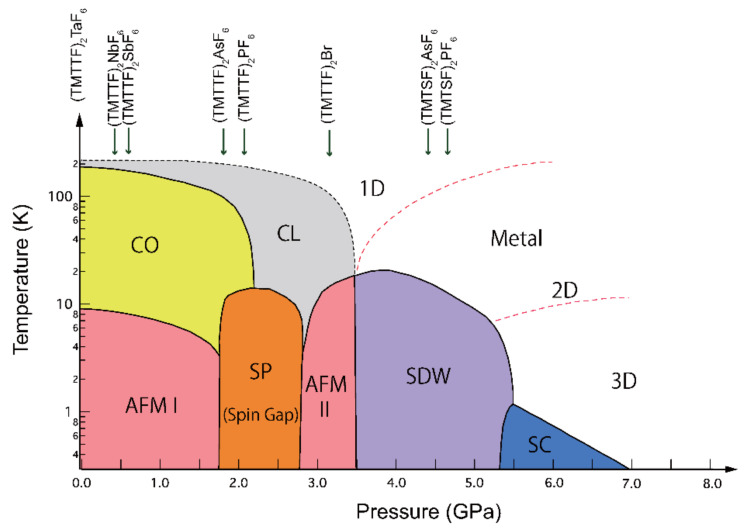
Electronic temperature-pressure (*T*-*P*) diagram for quasi-one-dimensional (Q1D) organic conductors (TMT*C*F)_2_*X* (*C* = S and Se), which start from the ground state of the (TMTTF)_2_TaF_6_ salt (CO state *T*_CO_ = 175 K and AFM *T*_AF_ = 9 K), suggested by Dressel et al. and Oka et al. This *T*-*P* diagram is depicted from the references [5,23,26,27,29,30,31,32]. The generalized *T*-*P* diagram (i.e., generalized electron correlation diagram) of (TMT*C*F)_2_*X* was first established by D. Jérome and coworkers in 1991 [1]. The abbreviations for described states in the diagram are CO (charge-ordering state), CL (charge-localized state), SP (spin-Peierls state), AFM (anti-ferromagnet, I: commensurate state, II: commensurate SDW), SDW (spin density wave), and SC (superconducting state).

The charge-ordering (CO) state was observed in the (TMTTF)_2_*X* series, except for (TMTTF)_2_ClO_4_, (TMTTF)_2_SCN, and (TMTTF)_2_I [19,20,21]. The CO transition of (TMTTF)_2_*X* is called a structureless transition since only a small change in the dimer position was observed by X-ray diffraction measurement [22]. The spatial charge disproportion on TMTTF molecules affects the spin state at low temperatures. In the case of (TMTTF)_2_*X,* which has an octahedron symmetry counter anion, the *X* = PF_6_ and AsF_6_ salts go into the SP phase from the CO phase, while the *X* = SbF_6,_ NbF_6_ [23], and TaF_6_ salts change to AFM I phase by cooling, depending on the CO pattern, dimensionality, and spin fluctuation [24,25]. Below 3 GPa pressure region, ^13^C NMR studies in *X* = SbF_6_ have revealed that the ground states vary from AFM I → spin gap → AFM II (C-SDW) (Figure 1) [26,27]. The ground state change from SDW to SC became decisive through the high-pressure resistivity measurement up to 10 GPa using a cubic anvil cell (CAC); the superconducting phase of (TMTTF)_2_SbF_6_ was observed as an anomalously wide pressure range (5.4 < *P* < 9 GPa) under limited temperature above 1.8 K [9].

(TMTTF)_2_TaF_6_ has the largest cell volume in the (TMT*C*F)_2_*X* series, with a *cs* anion. The one-dimensional character was confirmed by the result of the single-crystal X-ray diffraction analysis [28]. To understand the electron correlation, confirmations of the existence of the SC state and the pressure-dependent ground-state change toward the SC state in (TMTTF)_2_TaF_6_ are necessary. In this paper, we investigated the resistivity behaviors of (TMTTF)_2_TaF_6_ under high pressures up to 8 GPa and compared it to those for other TMTTF compounds (*X* = PF_6_, AsF_6,_ and SbF_6_).

## 2. Experimental Section

The sample preparation of (TMTTF)_2_TaF_6_ was detailed in Ref. [28]. The single crystal (TMTTF)_2_TaF_6_ is a black needle type; the sample size used for resistivity measurement was 800 μm × 15 μm × 10 μm. The high-pressure resistivity measurement was performed by using a CAC apparatus at the Institute for Solid State Physics (ISSP), The University of Tokyo. The resistivity of (TMTTF)_2_TaF_6_ was measured along the *a*-axis (crystal growing direction) by a conventional four-probe method. The four Au electric terminals (ϕ 10 μm Au wire) were in contact with carbon paste on the sample surface and the ends of Au wires were relayed to ϕ 15 μm Pt wires with Au paste to avoid wire cutting during the application of high pressures. The four relayed Pt wires are fixed with a small piece of paper by using epoxide resin adhesives to prevent sample breaking by the shock when the high pressure was applied. The sample with electrodes was sealed in a small Teflon cell (ϕ 2.0 mm) with the pressure medium Fluorinert 70:77 (1:1) mixture and then wrapped with a cubic MgO gasket (6 mm squares), which also served as the second pressure medium. The four Pt electric leads outside of the Teflon cell were wired with thin Au ribbons on the MgO gasket. The MgO gasket was isotopically compressed up to 250 tons by Tungsten carbide anvils, which also play a role in four-wire electronic contact materials. The CAC can generate a high-quality steady hydrostatic pressure by controlling a constant load system up to 12 GPa. The temperature range for this measurement was from 1.8 K to 295 K and the resistance value of (TMTTF)_2_TaF_6_ was recorded upon heating. External pressure was applied on (TMTTF)_2_TaF_6_ between 0 GPa and 1.4 GPa; the resistance varied due to unsteady attachment between the gasket and CAC anvils. At 1.5 GPa, the MgO gasket was compressed enough to achieve steady resistance of the TaF_6_ salt. Therefore, we did not measure the resistivity at 0 GPa; the measurement starts from 1.5 GPa. The resistivity along the *a*-axis was normalized by using the length and the surface values at room temperature.

## 3. Results and Discussion

At ambient pressure, the CO phase transition temperature (*T*_CO_ = 175 K) of *X* = TaF_6_ is the highest in the TMTTF series with octahedron monovalent anion. The CO transition temperature increases with increasing the octahedron anion size, PF_6_ (70 K), AsF_6_ (102 K) SbF_6_ (154 K), and NbF_6_ (165 K) [23]. The stoichiometry-controlled salt (TMTTF)_2_[AsF_6_]_1-*x*_[SbF_6_]*_x_* (*x*~0.3) has a higher *T*_CO_ of 120 K, which is between the temperatures of the AsF_6_ and SbF_6_ salts, and the characteristic SP magnetic susceptibility behavior is suppressed at a low temperature [33]. It was reported that the transition between SP and AFM I states occurs at *x*~0.5 chemical pressure [34], while the pressure experiment on (TMTTF)_2_SbF_6_ revealed that the AFM I state changed to a spin gap state due to the external pressure of 0.5 GPa, and the CO state becomes unclear above 0.5 GPa. [26]. (More details for the pressure effect on the CO state in *X* = AsF_6_ and SbF_6_ are in Ref [35]). The CO state in the TaF_6_ salt has a -O-o-O-o- pattern (O: charge-rich site, o: charge-poor site) as in the SbF_6_ salt, which coexists in the AFM I phase at ambient pressure [27,32]. The temperature-dependent magnetic susceptibility shows a characteristic one-dimensional decrease from 300 K to 50 K, and a rapid decrease was observed around 30 K. (TMTTF)_2_TaF_6_ undergoes an anti-ferromagnetic transition at 9 K [27].

**Figure 2:**
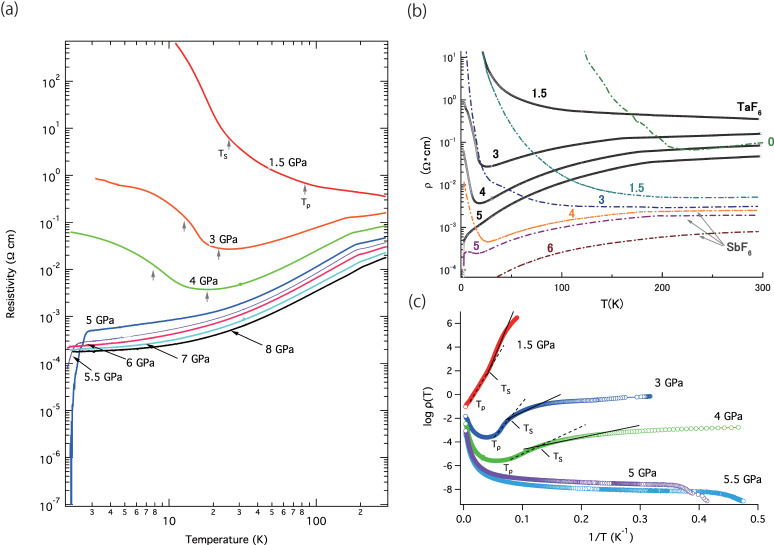
(**a**) Temperature dependence of the resistivity of (TMTTF)_2_TaF_6_ under several pressures (from 1.5 GPa to 8 GPa). The external pressure was applied by using the CAC pressure generator, which can compress the sample with liquid pressure-transmitted medium in a Teflon cell-surrounded MgO gasket from six directions simultaneously, using an external automatic 250-ton load piston. The temperature range for the measurement was from 1.8 K to room temperature. The resistivity along the *a*-axis direction was measured by the four-probe method; the resistivity data were recorded on heating procedure by slow temperature control. (**b**) Resistivity versus temperature for (TMTTF)_2_TaF_6_ (black lines) and (TMTTF)_2_SbF_6_ (dash-dot lines). The resistivity of (TMTTF)_2_SbF_6_ is reproduced by Ref. [9]. The ground states for both samples are CO (*T*_CO_ = 175 K for *X* = TaF_6_ and 154 K for *X* = SbF_6_) and AFM (anti-ferromagnetism, *T*_AF_ = 9 K for *X* = TaF_6_ and 8 K for *X* = SbF_6_). (TMTTF)_2_TaF_6_ shows higher resistivity than (TMTTF)_2_SbF_6_. (**c**) $\log\rho \left(T\right)$ versus 1T plot. *T*_𝜌_ and *T*_S_ are defined as points of the first slope change and the second change in $\log\rho \left(T\right)$ -1T plot.

Figure 2a shows the temperature-dependent resistivity of (TMTTF)_2_TaF_6_ in the *a*-axis direction under pressures from 1.5 GPa to 8 GPa. For the measurement, the initial temperature was set to around 1.8 K by cooling from room temperature, and then the temperature was gradually heated. Generally, the metallic conductivity of the TMTTF series becomes unstable due to the charge localization at *T*_𝜌_ in the high-temperature region. For the TaF_6_ salt, *T*_𝜌_ is reported as ~200 K, which is quite close to *T*_CO_ = 175 K. The resistivity at room temperature is 0.1 Ω·cm [32]. In the case of our pressure measurement, the resistivity at 1.5 GPa was 0.35 Ω·cm at room temperature, whose value was two orders higher than that (0.005 Ω·cm) of (TMTTF)_2_SbF_6_ (Figure 2b). A possible cause of the high resistivity value at 1.5 GPa is considered as a partial remnant charge localization caused by interfering with smooth volume compression and/or exceeding the anisotropic crystal deformation by a large TaF_6_ anion under low pressures.

**Figure 3:**
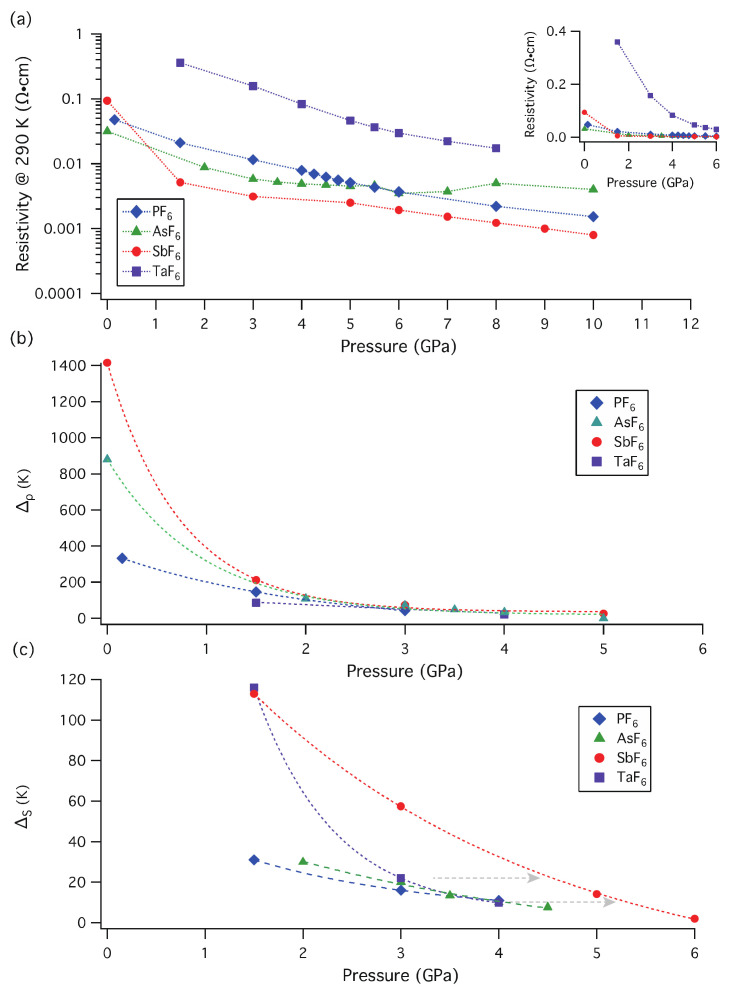
(**a**) Resistivity at 290 K versus pressure plots for (TMTTF)_2_*X* (*X* = PF_6_, AsF_6_, SbF_6,_ and TaF_6_) obtained using the CAC pressure generator (single logarithmic plot). (Inset) Enlarged resistivity-pressure graphs below 6 GPa. Pressure-dependent activation energy (**b**) Δ_𝜌_ and (**c**) Δ_S_ for (TMTTF)_2_*X* (*X* = PF_6_, AsF_6_, SbF_6_, and TaF_6_) obtained by fitting of logρTvs 1T plots. Dot lines are a guide for the eyes. The resistivities $\rho \left(T\right)$ along *a*-axis are regulated by the sample size at ambient pressure of 300 K. To compare with the data for (TMTTF)_2_TaF_6_, we reproduced the resistivity-pressure plot Δ_𝜌_ and Δ_S_ for *X* = PF_6_, AsF_6_, and SbF_6_ using references [7,8,9].

Figure 3a shows the resistivity value at 290 K under several pressures for (TMTTF)_2_*X* (*X* = PF_6_, AsF_6_, SbF_6,_ and TaF_6_) by using the CAC pressure generator. The pressure-dependent resistivities of *X* = PF_6_, AsF_6,_ and SbF_6_ were plotted with the data in Refs. [7,8,9]. The resistivity rapidly decreases up to 5 GPa and the value becomes almost unchanged beyond 6 GPa, although the resistivities for (TMTTF)_2_*X* (PF_6_, AsF_6_, and SbF_6_) are almost suppressed below 2 GPa (Figure 3a inset). It should be noted that the resistivity of the SbF_6_ salt is the lowest in the (TMTTF)_2_*X* series from 1.5 GPa to 10 GPa.

For the resistivity of the TaF_6_ salt at 1.5 GPa, we observed the drastic resistivity drop up to 100 K upon heating. After the temperature reached 100 K, the resistivity has a gradual incline (Figure 2b). The activation energies of Δ_𝜌_ and Δ_S_ were obtained from a simple formula, $\log\rho \left(T\right)=\frac{\Delta }{T}+\log\rho_{0}$. The inclination changes at a higher temperature and a second inclination change at a lower temperature correspond to *T*_𝜌_ and *T*_S_ in Figure 2c, in $\log\rho \left(T\right)$-1T plot. Here, the sample size reduction caused by thermal expansion and pressure effects is not taken into account for the $\log\rho \left(T\right)$ calculation. Figure 3b,c show the pressure-dependent activation energies (Δ_𝜌_ and Δ_S_) for (TMTTF)_2_*X* (*X* = PF_6_, AsF_6_, SbF_6_ and TaF_6_). It should be noted that the Δ_𝜌_ value in Figure 3b includes both activation energies coming from CO and Mott localization. The CO gap $\Delta_{\mathrm{CO}}$ has been discussed in several reports by resistivity measurements [20,32,33], using the formula $\Delta_{\mathrm{CO}}=\sqrt{\Delta {\left(T\right)}^{2}-\Delta^{2}\left(T_{\mathrm{CO}}\right)}$ (here $\Delta \left(T\right)=T\ln\rho$ in the CO state and $\Delta^{2}\left(T_{\mathrm{CO}}\right)$ is the value above $T_{\mathrm{CO}}$). Each $\Delta_{\mathrm{CO}}$ gap at ambient pressure is ~560 K for TaF_6_, ~500 K for SbF_6_, 430 K for [AsF_6_]_1-*x*_[SbF_6_]*_x_* (*x* ~0.3), ~315 K for AsF_6_, and ~217 K for PF_6_, which increases proportionally to *T*_CO_ [32,33]. $\Delta_{\mathrm{CO}}$ values are less than half of the obtained Δ_𝜌_ values at 0 GPa and the order of magnitude of (TMTTF)_2_*X* is Δ_𝜌_ (SbF_6_) > Δ_𝜌_ (AsF_6_) > Δ_𝜌_ (PF_6_), which is different from Δ_co_ (TaF_6_) > Δ_co_ (SbF_6_) > Δ_co_ (AsF_6_) > Δ_co_ (PF_6_). For the obtained data of high-pressure measurements for the TaF_6_ salt, the extraction of the $\Delta_{\mathrm{CO}}$ value at around 1.5 GPa is already unresolved due to an unclear transition at *T*_CO_.

The Δ_𝜌_ and Δ_S_ at 1.5 GPa for (TMTTF)_2_TaF_6_ are 117 K (*T*_𝜌_~90 K) and 116 K (*T*_S_~20 K), respectively, which correspond to the values at about 2.1 GPa and 1.5 GPa for those of (TMTTF)_2_SbF_6_, respectively. The Δ_𝜌_ becomes comparable to those of other (TMTTF)_2_*X* salts near 2.0 GPa since the CO state of the TaF_6_ salt already vanishes, then Δ_𝜌_ values coincide with almost constant values in (TMTTF)_2_*X* salts above 3 GPa. Meanwhile, activation energy Δ_S_ corresponds to a spin-related transition (SP, AFM II, and SDW), according to the similar analysis of (TMTTF)_2_*X* [2,3,7,8,9]. With increasing applied pressures, high resistivity at low temperatures dramatically drops and the metallic behavior becomes dominant above 40 K upon heating (see Figure 2b). At 3 GPa, the resistivity at room temperature still shows a high value of 0.3565 Ω·cm; the minimum drop in the resistivity curve appears at around 20 K in the resistivity-temperature curve, written in both logarithmic axes. The respective activation energies Δ_S_ at 3 GPa and 4 GPa are 22 K (*T*_S_ = 13 K) and 10 K (*T*_S_ = 8 K), respectively, which correspond to the activation energies observed in the *X* = SbF_6_ salt at ~4.6 GPa and 5.3 GPa, respectively (see Figure 3c). It should be noted that the AFM II (C-SDW) and SDW (I-SDW) transition temperatures are *~*15 K (at 0 ≤ *P* < 0.3 GPa) and ~23 K (at 0.3 < *P* ≤ 0.75 GPa) in (TMTTF)_2_Br [12]. Here, the resistivity of the TaF_6_ salt between 200 K and 300 K at 4 GPa becomes almost equal to that of *X* = SbF_6_ at room temperature (see Figure 2b).

**Figure 4:**
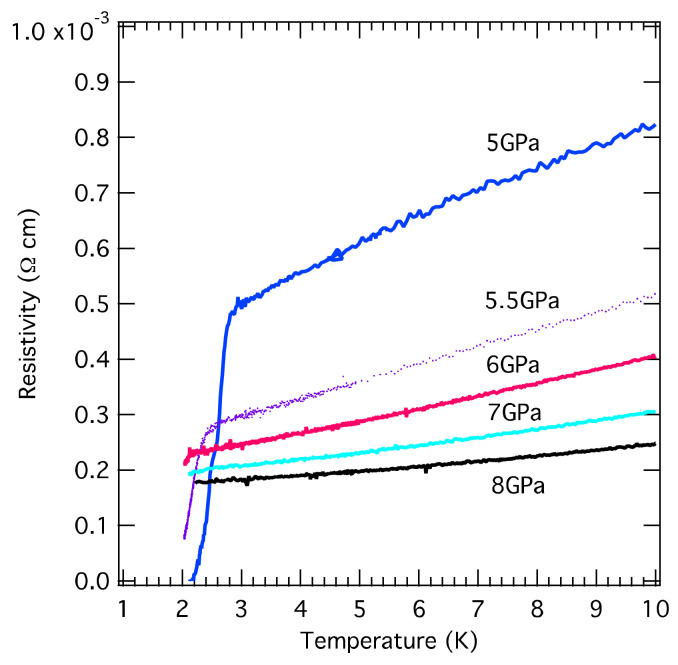
Temperature-dependent resistivity from 1.8 K to 10 K for (TMTTF)_2_TaF_6_ under various pressures. The lowest-achieving temperature is 1.8 K for the resistivity measurement using the CAC pressure generator. Superconducting behavior is observed around 2 K in the narrow pressure region, between 5 GPa and 6 GPa. Above 7 GPa, the superconducting state is not observed in the measured temperature range; the resistivity linearly increases as temperature increases.

At 5 GPa, the zero-resistivity is observed due to an occurrence of a pressure-induced superconductivity transition at 2.83 K. It should be noted that the value of resistivity (0.047 Ω cm) at 300 K is one order higher than that of the SbF_6_ salt and the zero-resistivity was observed using the CAC apparatus in (TMTTF)_2_*X* series except for the X = SbF_6_ salt [7,8,9]. Figure 4 displays the temperature-dependent resistivity from 5 GPa to 8 GPa at low temperatures (between 1.8 K and 10 K). The superconductivity phase exists in a quite short pressure region of 5 ≤ *P* ≤ 6 GPa; the shape of the SC phase is completely different from the (TMTTF)_2_SbF_6_ salt (5.4 ≤ *P* ≤ 9 GPa). The maximum *T*_C_ is 2.8 K at 5 GPa, the superconducting temperature shifts to a slightly lower temperature with increasing pressure, and then the SC state almost disappears at 6 GPa. Above 7 GPa, only metallic behavior is observed. By estimating the power of temperature (T) with $\rho \left(T\right)\;\sim \;T^{\alpha }$ between 6 GPa and 8 GPa, the *α* increases linearly as pressure increases and reaches ~1.5 at 8 GPa.

**Figure 5:**
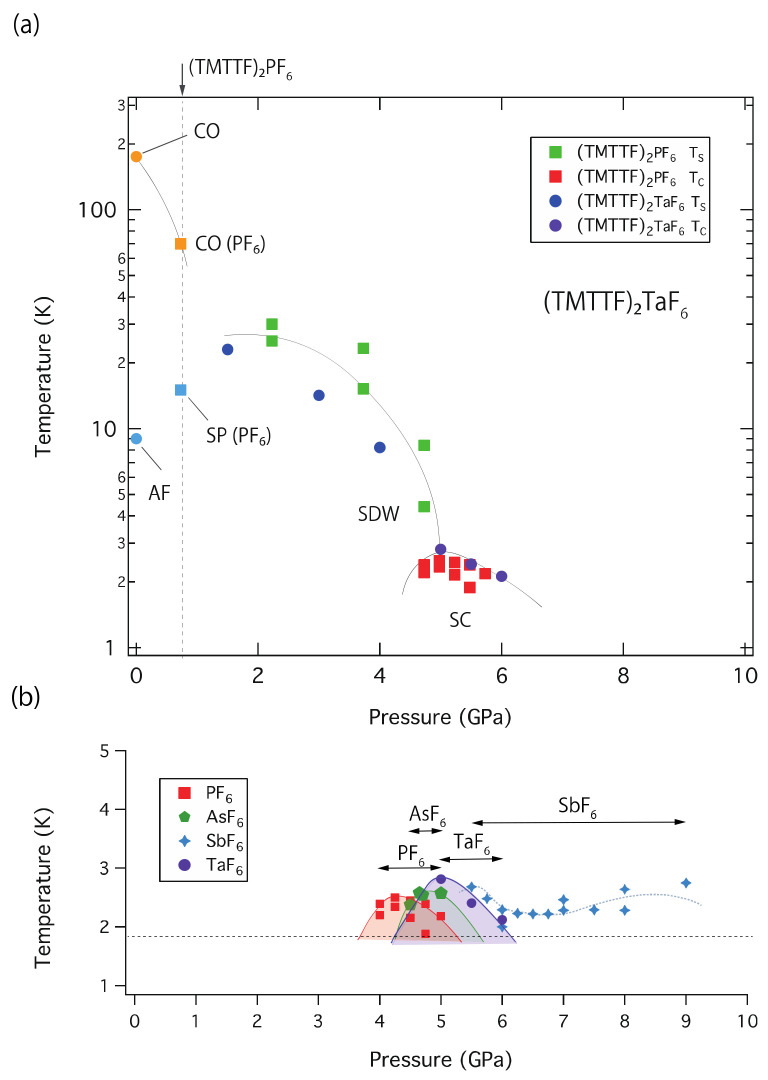
(**a**) Temperature-pressure (*T-P*) diagram of (TMTTF)_2_TaF_6_. The denoted ground states were obtained by referring to the electronic correlation of the PF_6_ salt. The offset pressure between the SC (superconducting) phases of PF_6_ and TaF_6_ is estimated at 0.75 GPa by adjusting the pressures, at which the highest temperatures of the SC were observed in TaF_6_ and PF_6_ salts. (**b**) Superconducting transition temperature versus applied pressures in (TMTTF)_2_*X* for indicating the pressure region of superconducting phases observed by the resistivity measurements using the CAC pressure generator (*X* = PF_6_ [7], AsF_6_ [8], SbF_6_ [9], and TaF_6_ (this work)). A horizontal dot line indicates the lowest temperature limit of 1.8 K when high-pressure resistivity measurements were carried out by the CAC.

The main physical parameters, maximum superconducting transition temperature *T*_C_ and the pressure *P*_C_, activation energy Δ_S_ at 3 GPa, lattice parameters along the *a*-axis, volumes, and ground states at ambient pressure for (TMTTF)_2_*X* (PF_6_, AsF_6_, SbF_6_, and TaF_6_) are listed in Table 1. The *T-P* diagram of (TMTTF)_2_TaF_6_ based on the result of this resistivity measurement is described in Figure 5, in which the electron-correlation change is referred to in the data of (TMTTF)_2_PF_6_. This phase diagram is obtained by adjusting both pressure points of TaF_6_ and the PF_6_, where the maximum SC transition temperatures (*T*_C_ (PF_6_) and *T*_C_ (TaF_6_)) were recorded. The ground states of the TaF_6_ salt vary in CO/AFM I -(SP)- SDW (C-SDW: commensurate spin density wave and I-SDW: incommensurate spin density wave) and SC as referred to in reports [1,2] and the previous studies [7,8,9]. In this case, the chemical pressure between the PF_6_ and TaF_6_ salts is roughly estimated as 0.75 GPa. Due to the fact that the ground state change at a lower pressure in the TaF_6_ salt has not yet been proven, the pressure range on the lower pressure side of Figure 1 differs from that of this *T*-*P* phase diagram.

The emergence of the SC phase in the narrow pressure region for the TaF_6_ salt is similar to those observed in the PF_6_ and AsF_6_ salts; however, the SC phase of the SbF_6_ salt is observed over a wide pressure range (see Figure 5b). The reason is probably attributed to the difference in structural compression sensitivity corresponding to the dimensionality by applied pressure and thermal expansion upon cooling. The structural investigation and the DFT calculations indicated that pressure (~2.7 GPa) and a lower temperature increase two-dimensionality in *X* = PF_6_ and SbF_6_ [36,37]. It was reported that, in the structure of (TMTTF)_2_PF_6_ under high pressure, the space group $P\bar{1}$ remains up to 8 GPa and a pressure-induced structural transition (triclinic → monoclinic phase transition) occurs above 8.5 GPa [38]. The lattice *a* is dramatically compressed to approximately 12.5% and then the total unit cell volume shrinks by about 27.5% by external pressure up to 8 GPa. At the SC-appearing pressure (~4.3 GPa), the compressed lattice and volume where the SC can be observed are *a*(PF_6_) ~ 6.44 Å and *V*(PF_6_) ~ 540 Å^3^, which are approximately 90% and 80% of the values at ambient pressure, respectively (see Table 1). Considering a simple estimation, since the actual pressure of the TaF_6_ salt shifts by 1 GPa to the negative pressure side of that of the PF_6_ salt, the volume of TaF_6_ would be compressed to approximately 22.5% by *V*(TaF_6_) ~ 547 Å^3^ to appear in the SC phase. Unfortunately, the bulk modulus and thermal expansion of *X* = SbF_6_ and TaF_6_ under high pressure are unknown. High pressure structural investigations in *X* = SbF_6_ and TaF_6_ are necessary to understand not only the origin of the high resistivity of TaF_6_ but also the narrower SC phase compared to that of the SbF_6_ salt.

## 4. Conclusions

We measured resistivity for (TMTTF)_2_TaF_6_ under high pressure (up to 8 GPa) using a CAC pressure apparatus that can generate hydrostatic pressure. (TMTTF)_2_TaF_6_ has charge-ordering (*T*_CO_ = 175 K) and anti-ferromagnetic (*T*_AF_ = 9 K) states at ambient pressure. At 3 and 4 GPa, the growth of resistivity was observed at low temperatures due to spin-related transition; SDW (C-SDW and/or I-SDW) is predicted from our T-P phase diagram, as observed in (TMTTF)_2_X (*X* = PF_6_, AsF_6_, and SbF_6_). With increasing applied pressure, a superconducting (SC) state appears at 5 GPa. The *T*_C_ of (TMTTF)_2_TaF_6_ records the highest SC temperature of 2.8 K (at 5 GPa) in the (TMTTF)_2_*X* series. However, the SC phase is observed in the short pressure region between 5 GPa and 6 GPa above ~2 K. From the results of high-pressure resistivity measurements with the CAC pressure generator for the Q1D organic conductor (TMTTF)_2_*X* (*X* = PF_6_, AsF_6_, SbF_6,_ and TaF_6_), the generalized TMT*C*F *T-P* diagram could be extended by confirming the SC phase in TaF_6_ salt with a negative offset pressure of Δ*P*~0.75 GPa when the pressures at maximum SC temperature were compared between the PF_6_ and the TaF_6_ salts. High-pressure X-ray structural measurement is future work required to reveal the different appearances of SC phase shapes between TaF_6_ and SbF_6_ salts under high pressure.

## Figures and Tables

**Table 1:** Physical properties of (TMTTF)_2_*X* (*X* = PF_6_, AsF_6_ SbF_6_, and TaF_6_). *T*_CO_, activation energies for Δ_S_ at 3 GPa, lattice parameters and volumes at ambient temperature, superconducting (SC) temperature *T*_C_ and the observed pressure, and ground states at low temperature (0 GPa). Δ_S_, *T*_C,_ and pressure region for SC were obtained using resistivity measurements with the CAC pressure generator.

(TMTTF)_2_*X*	*T*_CO_	Activation Energy 3 GPa Δs [K]	Lattice Parameter a* [Å]	V [Å^3^] at Room Temperature *	Superconducting Temperature *T*_C_ [K]	Pressure at Maximum *T*_C_ [K] (Pressure Region for SC Phase) **	*T*_SP_ [K]	*T*_AF_ [K]
PF_6_	70	16	7.172(11)	676.6	2.5	4.3 (4.0 ≤ *P* ≤ 5.0 GPa)	15	
AsF_6_	102	17	7.1662(4)	686.15	2.6	5.0 (4.5 ≤ P ≤ 5.0 GPa)	14	
SbF_6_	154	57	7.1796(11)	702.93	2.8	6.0, 9.0 (5.4 ≤ *P* ≤ 9.0 GPa)		8
TaF_6_	175	22	7.1862(11)	706.52	2.8	5.0 (5.0 ≤ *P* ≤ 6.0 GPa)		9

* Lattice parameters and volumes refer to Ref. [28]. ** The temperature region for the resistivity measurement by the CAC was from 1.8 K to 300 K.

## Data Availability

Not applicable.
